# Microencapsulated *Bifidobacterium bifidum* and *Lactobacillus gasseri* in Combination with Quercetin Inhibit Colorectal Cancer Development in Apc^Min/+^ Mice

**DOI:** 10.3390/ijms22094906

**Published:** 2021-05-05

**Authors:** Iván Benito, Ignacio J. Encío, Fermín I. Milagro, María Alfaro, Ana Martínez-Peñuela, Miguel Barajas, Florencio Marzo

**Affiliations:** 1 Laboratory of Animal Physiology and Nutrition, School of Agronomy, Public University of Navarre, Campus Arrosadia, 31006 Pamplona, Spain; ibenito.penalver@gmail.com (I.B.); malfaro.larraya@gmail.com (M.A.); 2 Biochemistry Area, Department of Health Science, Public University of Navarre, 31008 Pamplona, Spain; ignacio.encio@unavarra.es; 3 Department of Nutrition, Food Sciences and Physiology, Center for Nutrition Research, University of Navarra, C/Irunlarrea 1, 31008 Pamplona, Spain; fmilagro@unav.es; 4 Centro de Investigación Biomédica en Red de la Fisiopatología de la Obesidad y Nutrición (CIBERobn), Instituto de Salud Carlos III, 28029 Madrid, Spain; 5 Laboratorio Martínez-Peñuela, C/Amaya, 31-Bajo, 31004 Pamplona, Spain; anapenuela@gmail.com

**Keywords:** quercetin, *Lactobacillus gasseri*, *Bifidobacterium bifidum*, energy expenditure, colon cancer, Wnt/β-catenin

## Abstract

Recent studies have suggested that flavonoids such as quercetin and probiotics such as *Bifidobacterium bifidum* (*Bf*) and *Lactobacillus gasseri* (*Lg*) could play a relevant role in inhibiting colon cancer cell growth. Our study investigated the role of dietary supplementation with microencapsulated probiotics (*Bf* and *Lg*) along with quercetin in the development of mouse colorectal cancer (CRC). Methods: Adenomatous polyposis coli/multiple intestinal neoplasia (Apc^Min/+^) mice were fed a standard diet or the same diet supplemented with microencapsulated probiotics (*Bf* and *Lg* strains, 10^7^ CFU/100 g food) or both probiotics strains plus microencapsulated quercetin (15 mg/100 g food) for 73 days. Changes in body and organ weights, energy metabolism, intestinal microbiota, and colon tissue were determined. The expression of genes related to the Wnt pathway was also analyzed in colon samples. Results: Dietary supplementation with microencapsulated probiotics or microencapsulated probiotics plus quercetin reduced body weight loss and intestinal bleeding in Apc^Min/+^ mice. An improvement in energy expenditure was observed after 8 weeks but not after 10 weeks of treatment. A supplemented diet with microencapsulated *Bf* and *Lg* reduced the number of aberrant crypt foci (ACF) and adenomas by 45% and 60%, respectively, whereas the supplementation with *Bf*, *Lg* and quercetin decreased the number of ACF and adenomas by 57% and 80%, respectively. Microencapsulated *Bf* and *Lg* in combination with quercetin could exert inhibition of the canonical Wnt/β-catenin signaling pathway in the colon of Apc^Min/+^ mice Conclusions: The administration of microencapsulated *Bf* and *Lg*, individually or in combination with quercetin, inhibits the CRC development in Apc^Min/+^ mice.

## 1. Introduction

Colorectal cancer (CRC) is the third most prevalent type of cancer in men, and second in women, and the fourth cause of mortality associated with cancer [1,2]. Epidemiological and experimental studies have shown a link between Western lifestyle and a higher risk of CRC. Thus, approximately 50% of colorectal cancer cases occur in developed countries [3,4]. Colon cancer development is usually associated with body weight loss, muscle and fat tissue loss, as well as changes in energy expenditure [5]. Gastrointestinal disorders, anorexia, taste changes, and early satiety have also been described [6]. These associated alterations are important because they cause a worsening of disease diagnosis and significantly affect the quality of life and life span [7].

Numerous studies have shown that diet plays an important role in the development of the disease. Diets rich in fatty meat and saturated fats increase the risk of developing CRC [3,4]. On the contrary, diets rich in flavonoids and dietary fiber are associated with a lower incidence of CRC [8,9,10,11].

Flavonoids are a diverse group of polyphenols found in foodstuffs like fruits, vegetables, wine, tea, and coffee. Quercetin is the most abundant flavonoid in the human diet. Several in vitro and in vivo studies have shown that quercetin has anti-inflammatory, antioxidant, anti-proliferative, and anticancer effects [12,13,14]. It is well known that dietary supplementation with quercetin (0.2% and 2%) has antitumor effects in animal models [15,16]. Besides, the intake of quercetin has a wide safety margin [17].

On the other hand, probiotics, such as lactic acid bacteria (LAB), can also play a beneficial role in the prevention of gastrointestinal disease, including CRC. Beneficial effects of probiotics are: Modulation of the intestinal microbiota, changes in the enteric physicochemical conditions, production of anti-tumorigenic or anti-mutagenic compounds, and improvement of the host’s immune response [18,19]. *Bifidobacterium bifidum* (*Bf*) and *Lactobacillus gasseri* (*Lg*) are lactic acid bacteria that have shown a preventive effect on the development of intestinal diseases, especially those involving inflammation [20,21]. The LAB are commonly used in human studies at a dose of 10^9^ CFU/day [22], although a dose of 10^11^ CFU/day of probiotics have been tested and did not show toxicity [23].

The use of quercetin and LAB as dietary supplements has some drawbacks to control the effective doses administered. Quercetin is metabolized in the gastrointestinal tract before entering the blood and internal organs [24,25]. On the other hand, the survival of probiotics decreases as a result of gastrointestinal transit [26]. A previous study from our laboratory, based on simulated digestion, found that chitosan and alginate microencapsulation protects Lactobacillus gasseri (Lg) and Bifidobacterium bifidum (Bf) during the gastrointestinal transit, significantly increased survival of these probiotics [27]. The microencapsulation process ensures that the probiotics used (*Lg* and *Bf*) are capable of passing the gastric barrier without being degraded, thus improving their survival and activity and, consequently, their therapeutic benefit. Microencapsulated *Lg* and *Bf* showed greater resistance to simulated gastric conditions (pH 2.0, 2 h) and bile solution (3%, 2 h), resulting in significantly (*p* < 0.05) improved survival when compared with free bacteria, as shown in a previously published article [27]. Regarding CRC, a study [25] showed that the oral administration of microencapsulated *Lactobacillus acidophilus* contributed to the stabilization of body weight, suppression of colon tumor incidence and multiplicity, and reduction of tumor size in spontaneously developed intestinal neoplasias in Apc^(Min/+)^ mice.

Thus far, the studies have focused on analyzing the preventive effect of probiotics and flavonoids against colon cancer independently. Although probiotics and prebiotics (fiber and resistant starch, and oligosaccharides such as inulin) have been found to exhibit a synergistic anticancer effect in colon cancer [28], there are no studies that analyze the combined effect of the coadministration of probiotics and polyphenols in the prevention of intestinal diseases, such as colon cancer.

The Apc^Min/+^ mice are considered a good model for gut cancer [29] because the mutation has similar effects to the APC mutation in humans [30]. This model allows studying the effects of genetics, chemical compounds, or diets on the incidence and development of CRC. A special feature of these mice is that their life span ranges from 120 to 160 days [31,32].

Our study examines the potential effect of dietary supplementation with microencapsulated probiotics with or without microencapsulated quercetin, in the prevention of CRC and the amelioration of signs associated with this disease in Apc^Min/+^ mice, by assessment of body weight, energy expenditure, respiratory quotient, organ weight, fat depots, intestinal bleeding, and histological alterations. We also examined the expression of genes involved in the Wnt signaling pathway that may explain the mechanism by which the combination of probiotics and quercetin exerts a beneficial effect in the prevention of CRC observed in Apc^Min/+^ mice.

## 2. Results

### 2.1. Food, Probiotics, and Quercetin Intake

According to the experimental design, food intake was determined throughout the time that the experimental protocol lasted. No significant differences were observed in food intake between the four groups during the experimental period (Table 1). The amounts of probiotics consumed by the E1 and E2 groups were similar, showing a mean probiotic intake of 2.31 × 10^4^ CFU/g bw/day. The amount of quercetin ingested by the E2 group was 17.25 μg/g bw/day. 

### 2.2. Body Weight

**Figure 1:**
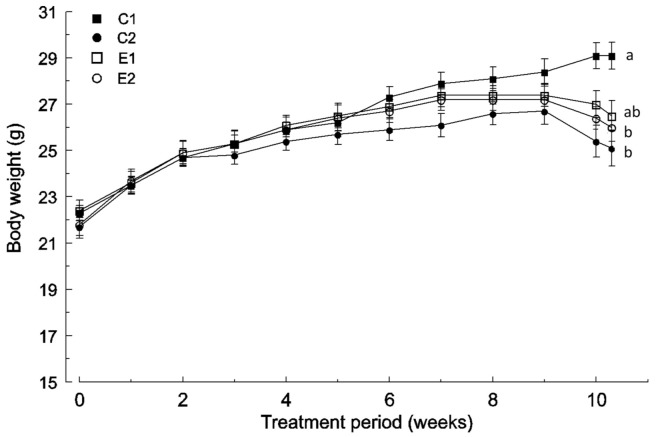
Body weight change in the four experimental groups (mean and SD, *n* = 12): *C1* wild-type mice fed a standard diet, *C2* Apc^Min/+^ fed a standard diet, E1 Apc^Min/+^ fed a standard diet supplemented with microencapsulated *Bf* and *Lg*, *E2* Apc^Min/+^ fed a standard diet supplemented with microencapsulated *Bf*, *Lg,* and quercetin. Mean values labeled with unlike letters are significantly different (*p* < 0.05; one-way ANOVA was used followed by Tukey’s post hoc test).

No differences were found in the body weight variable between the four experimental groups during the first 2 weeks of the experimental protocol. Since then, the C2 group showed a reduction in body weight growth in comparison with the wild-type that reached statistical significance (*p* <0.05) at week 10 (Figure 1). Nevertheless, the C2 group did not show significant differences with respect to E1 and E2 groups at any time.

Body weights of the E1 and E2 groups were similar to the wild-type (C1 group) until the 6th week, but a decrease was observed in the last weeks (Figure 1). The E2 group showed lower body weight (*p* < 0.05) than the wild-type (C1 group) from the 10th week (Figure 1). However, the E1 group did not show significant differences with respect to any group at any time point.

All the Apc^Min/+^ mice showed a reduction in body weight during the last weeks of the experimental time, but it was more pronounced in the non-supplemented C2 group (−5.84%) than in E1 (−3.20%) and E2 (−3.03%) groups (Figure 1).

### 2.3. Energy Metabolism

No statistical differences were found in the respiratory quotient (RQ) between the experimental groups at weeks 4 and 8. In the 10th week of treatment, the RQ was higher in the group C2 as compared to C1 and E2 groups (*p <* 0.05). The RQ of the E1 group was not significantly different with respect to the other groups, whereas the RQ of the group E2 resembled that of wild-type mice. 

All Apc^Min/+^ groups showed higher energy expenditure (EE) in the 4th week of treatment (*p <* 0.05) than wild-type mice, but no statistical differences were observed among them. Amelioration in EE was observed in the 8th week of treatment since EE of the C1 group resembles that of E1 group (Table 2). Finally, in the 10th week, all Apc^Min/+^ groups showed lower energy expenditure than the wild-type but with no significant differences between them (Table 2).

### 2.4. Tissue and Organ Weight

All Apc^Min/+^ mice, especially the C2 group, showed lower gastrocnemius muscle weight compared to wild-type animals (C1 group) (Table 3). However, these differences did not reach statistical significance. Similarly, no significant differences were found regarding thymus weight, although it tended to be lower in the three groups of Apc^Min/+^ mice, especially in group C2.

Liver weight was higher (*p <* 0.05) in the groups C2 and E1 than in wild-type mice (Table 3). In the case of E2, no statistical differences were found with respect to the other groups.

Spleen weight was higher in all Apc^Min/+^ groups (*p <* 0.05) than in wild-type mice (Table 3). Nevertheless, no significant differences in the weight of the spleen were observed among the groups of Apc^Min/+^ mice. The increase of spleen weight was lower in the case of the animals fed with both supplemented diets (E1 and E2).

The changes in body fat depots were quantitatively lower in all Apc^Min/+^ groups (C2, E1, and E2 groups) as compared to wild-type (C1 group), which was especially significant for the group C2 (Table 3). This decrease was more relevant in the perirenal and epididymal fat deposits, which were lower (*p <* 0.05) in the three groups of Apc^Min/+^ mice. Nevertheless, this reduction was smaller in the groups fed the supplemented diets (E1 and E2).

### 2.5. Faecal Occult Blood Detection

**Figure 2:**
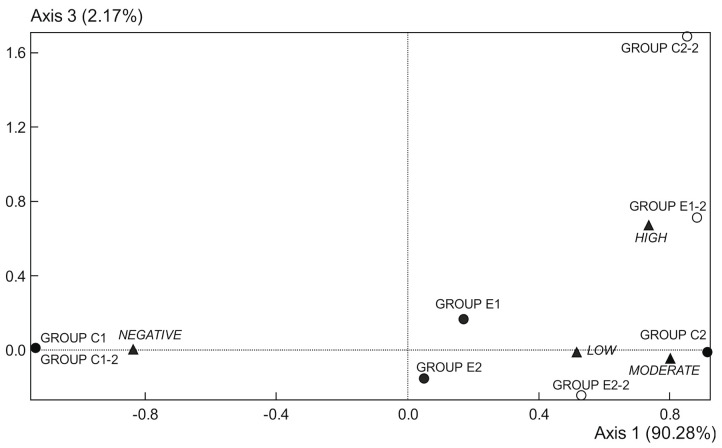
Correspondence analysis scores; plot of occult fecal blood data from 1st week (●) and 9th week (○). Data points represent replicates of the experimental groups (*C1*, *C2*, *E1*, and *E2*), and intensities of staining (▲) (from absence to very intense). *C1* wild strain fed a control diet, *C2* Apc^Min/+^ fed a control diet, *E1* Apc^Min/+^ fed a diet supplemented with microencapsulated *Bf* and *Lg*, *E2* Apc^Min/+^ fed a diet supplemented with microencapsulated *Bf*, *Lg,* and quercetin.

Data of fecal occult blood (FOB) were determined in weeks 1 and 9 (Table 4 and Figure 2). All samples from C1 group showed an absence of FOB in both weeks. In the first week, the C2 group showed a moderate presence of FOB, while both E1 and E2 groups were characterized by lack and poor blood in fecal samples. In the 9th week, the C2 group showed a high presence of blood. The E1 group was now characterized by moderate to high blood, and the E2 group by low to moderate blood. Thus, both supplemented diets reduced intestinal bleeding with respect to the C2 group in the two studied weeks, being the supplementation with microencapsulated probiotics and quercetin (E2 group) more effective for this purpose (Figure 2 and Table 4).

### 2.6. Microbiological Analysis

**Figure 3:**
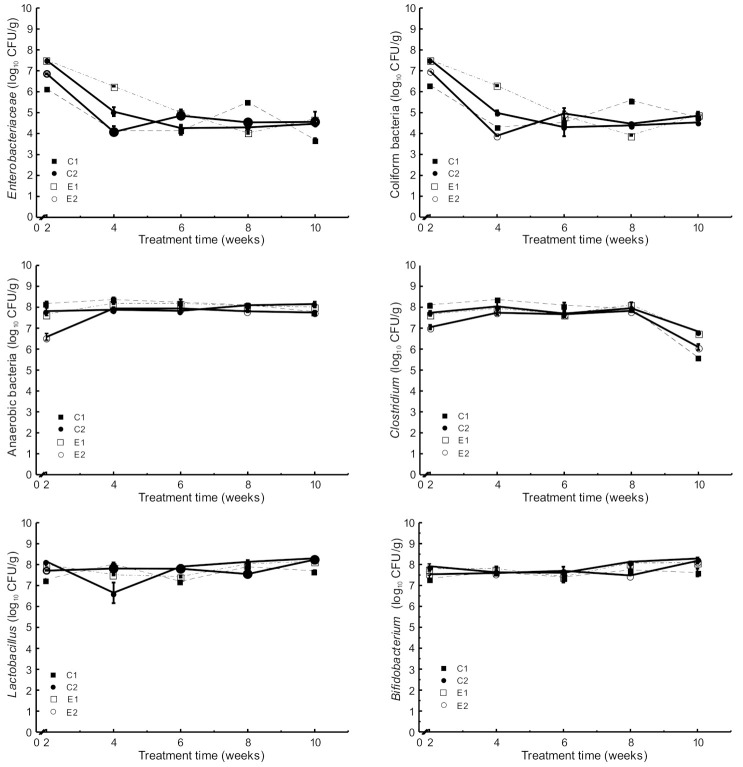
Microbial counts in stool samples collected during the experimental period (mean and SD, *n* = 6): *C1* wild-type mice fed a standard diet, *C2* Apc^Min/+^ fed a standard diet, *E1* Apc^Min/+^ fed a standard diet supplemented with microencapsulated *Bf* and *Lg*, *E2* Apc^Min/+^ fed a standard diet supplemented with microencapsulated *Bf*, *Lg,* and quercetin.

The results of the microbiological analyses throughout the experimental period showed no significant differences in the different populations of microorganisms analyzed among the four experimental groups (Figure 3).

**Figure 4:**
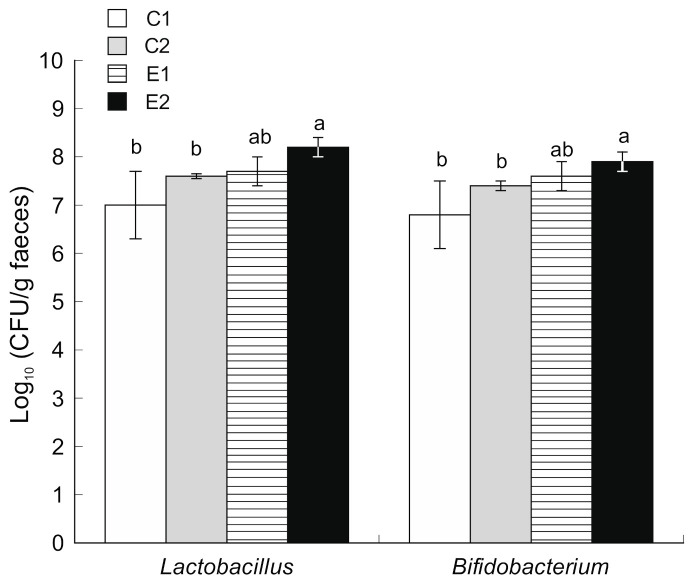
Microbial counts of *Lactobacillus* and *Bifidobacterium* in caecum faecal samples obtained after sacrifice (mean and SD, *n* = 12). *C1* wild-type mice fed a standard diet, *C2* Apc^Min/+^ fed a standard diet, *E1* Apc^Min/+^ fed a standard diet supplemented with microencapsulated Bf and Lg, *E2* Apc^Min/+^ fed a standard diet supplemented with microencapsulated *Bf*, *Lg* and quercetin. Bars with different letters indicate statistically significant differences (*p* < 0.05; one-way ANOVA was used followed by Tukey’s post hoc test).

The count of *Enterobacteriaceae* in stool samples was decreased from 10^7^ CFU/g (2nd week) to 10^4^ CFU/g (10th week) (Figure 3). Similarly, the count of Coliform bacteria was decreased from 10^7^ CFU/g (2nd week) to 10^4^ CFU/g (10th week). No other significant differences were observed between the four experimental groups. However, animals supplemented with microencapsulated probiotics and quercetin (E2) showed increased counts (*p* < 0.05) of *Bifidobacterium* and *Lactobacillus* population in the caecum content compared to both C1 and C2 (Figure 4).

### 2.7. Histological Analysis 

Samples of colon obtained from C1 group (wild-type mice fed with a control diet) did not present ACF or adenomas. In contrast, C2 samples (Apc^Min/+^ fed with the control diet) presented an average of 17.75 ACF and 1.25 adenomas per mouse. Dietary supplementation with microencapsulated probiotics (E1) or microencapsulated probiotics and quercetin (E2) significantly reduced the number of ACF by 45% and 57% compared to C2, respectively. The supplementation also reduced the number of adenomas by 60% in the E1 group and 80% in the E2 group compared to C2 (Table 5).

**Figure 5:**
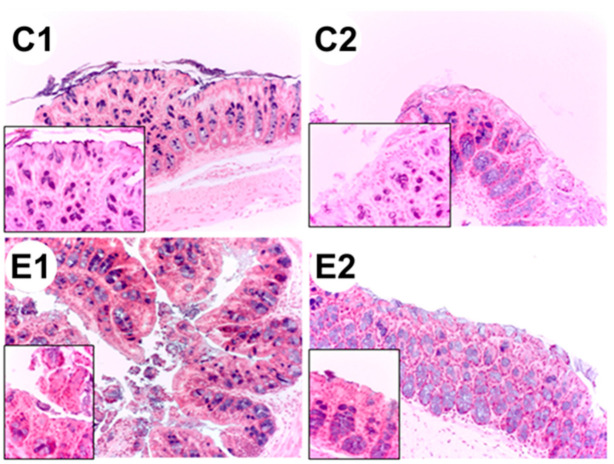
Histological sections of the colon from each experimental group: **C1** wild-type mice fed a standard diet, **C2** Apc^Min/+^ fed a standard diet, **E1** Apc^Min/+^ fed a standard diet supplemented with microencapsulated *Bf* and *Lg*, **E2** Apc^Min/+^ fed a standard diet supplemented with microencapsulated *Bf, Lg,* and quercetin. The images were taken at 20× and at 63× (left lower panel, increased epithelial).

Representative images of colon histological sections stained with hematoxylin-eosin obtained from colon samples of Apc^Min/+^ mice (C2 group) showed dysplasia and hypertrophy of the Lieberkühn crypts (Figure 5).

### 2.8. Supplemented Diet Alter the Expression of Genes Involved in the Wnt Signaling Pathway

Due to the importance of the abnormal regulation of the Wnt/β-catenin signaling pathway in the development of colorectal cancer, we decided to analyze whether supplemented diets modulated the expression of different genes of this signaling pathway in the colon. With this purpose, animals were fed as described above, and, after 73 days of treatment, the expression level of 84 genes related to the Wnt/β-catenin signaling pathway was determined by qPCR (RT2 profiler Wnt Signaling Pathway PCR Array PAMM-043Z, SA Biosciences) in distal colon samples. Obtained results are shown in Table 6 and Table 7. Only those genes whose expression augmented more than 2-fold or those whose expression was reduced by at least 40% were displayed.

**Figure 6:**
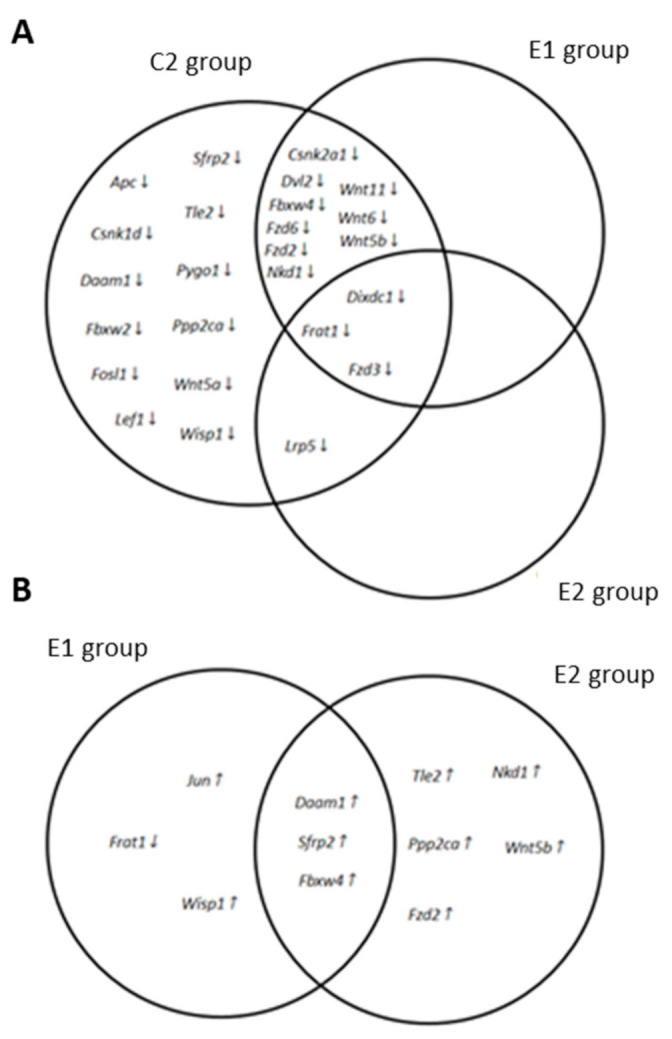
An overlapping pattern of genes of the Wnt signaling pathway modified in APC^Min/+^ mice fed with standard and supplemented diets. Edwards-Venn diagrams showing the genes overexpressed at least 2 times (up arrows) or downregulated by 50% (down arrows) in APC^Min/+^ mice fed with a standard diet (C2 group), fed with a (*Bf*+*Lg*) supplemented diet (E1 group), or fed with a (*Bf*+*Lg*+Q) supplemented diet (E2 group). (**A**) Genes modified in APC^Min/+^ mice as compared to wild-type mice. (**B**) Genes modified in APC^Min/+^ E1 and E2 groups fed with supplemented diets as compared to the APC^Min/+^ control group (C2).

As shown in Table 6, the expression of 25 genes (*Apc, Csnk1d, Csnk2a1, Daam1, Dixdc1, Dvl2, Fbxw2, Fbxw4, Fosl1, Frat1, Fzd2, Fzd3, Fzd6, Lef1, Lrp5. Nkd1, Ppp2ca, Pygo1, Sfrp2, Tle2, Wisp1, Wnt11, Wnt5a, Wnt5b, Wnt6*) was reduced to less than their halves in the APC^Min/+^ mice group fed with a standard diet as compared to the wild-type control group. However, no gene whose expression was increased at least twice as compared to wild-type was detected in the APC^Min/+^ mice. Table 6 also shows that some of the genes whose expression was downregulated in the APC^Min/+^ control group, including Apc, increased their expression level when mice were fed with supplemented diets. As a result, only 12 genes in the E1 group (*Csnk2a1, Dixdc1, Dvl2, Fbxw4, Frat1, Fzd2, Fzd3, Fzd6, Nkd1, Wnt11, Wnt5b, and Wnt6*) and 4 genes in the E2 group (*Dixdc1, Frat1, Fzd3, and Lrp5*) displayed expression levels smaller than 60% of those detected in the WT control group. Figure 6A summarizes the overlapping pattern of genes downregulated in the different APC^Min/+^ experimental groups.

Table 7 shows the fold change in the expression level of those genes whose expression varied in either of the experimental groups of APC^Min/+^ mice fed with a supplemented diet when compared to the APC^Min/+^ mice fed with a standard diet. Interestingly, only one gene (*Frat1*) showed lower expression levels in the APC^Min/+^ mice fed with supplemented diets than in the APC^Min/+^ control group. However, 5 genes in the E1 group (*Damm1, Fbxw4, Jun, Sfrp2, and Wisp1*) and 8 genes in the E2 group (*Daam1, Fbxw4, Fzd2, Nkd1, Ppp2ca, Sfrp2, Tle2 and Wnt5b*) increased their expression at least twice over the APC^Min/+^ control group (Table 7). As shown in Figure 6B, only 3 genes (*Damm1, Fbxw4, and Sfrp2*) augmented at least twice their expression level in APC^Min/+^ mice when fed with either of the supplemented diets.

## 3. Discussion

The present study demonstrates that the supplementation with microencapsulated *Bf* and *Lg* with or without microencapsulated quercetin could alleviate some signs associated with CRC development. The animal model chosen to study the colon cancer development process was the Apc^Min/+^ mouse, one of the animal models that best represent the initiation and progression of intestinal tumorigenesis. We also use a microencapsulation technology to protect active therapeutic agents during intestinal transit. Thus, the application of a microencapsulation system based on chitosan and alginate increased the survival of lactic acid bacteria used as probiotics and prevented the degradation and modification of quercetin due to the intestinal transit conditions [27]. The amounts of ingested probiotics were equivalent to recommended doses in humans (10^9^ CFU/day) [22]. Previous studies have reported the preventive effects of quercetin on the development of CRC using doses of quercetin from 0.2 to 2 mg/100 g diet [15,16]. The concentration used in our study was 0.015 mg/100 g diet mice, corresponding to 1.2 g/day of quercetin intake in humans.

CRC is often associated with uncontrolled body weight loss, reduced food intake, and altered energy expenditure [33]. In the present study, food intake did not show statistically significant differences between the experimental groups, whereas weight loss was slightly lower in the mice treated with the microencapsulated compounds. Uncontrolled body weight loss associated with cancer affects the life quality and life span of oncology patients [5,34]. In the Apc^Min/+^ mice, body weight loss was accompanied by a significant reduction (*p <* 0.05) in adiposity, and, similarly to weight loss, fat depletion was higher in the unsupplemented group. In addition, although other authors have reported muscle wasting in animals with cancer [35,36], in our study Apc^Min/+^ mice showed only a reduction in gastrocnemius muscle weight that was not statistically significant. These results show that microencapsulated probiotics and quercetin could alleviate the cachexia associated with colorectal cancer.

It is known that APC gene mutation causes splenomegaly and can also affect other organs, such as the thymus and liver [37,38]. In the present study, animals fed the supplemented diets (E1 and E2) showed a lower increase in spleen weight than those fed the control diet (C2), but this improvement was not statistically significant. In the same way, Apc^Min/+^ mice showed a significant increase of liver weight that was lower in the group fed microencapsulated probiotics and quercetin (E2), which remained similar to the wild-type (C1). The spread of tumor cells to the liver [39] and the development of fibrosis and fatty degeneration [40] could explain hepatomegaly.

As other authors have previously reported in Apc^Min/+^ mice [38], we have not detected a significant reduction in thymus weight. However, other studies in Apc^Min/+^ mice noticed atrophy of the thymus after 80 days of age [41].

Tumour development produces changes in energy expenditure that depends on the type of tumor [5,42]. Comparing the energy expenditure of Apc^Min/+^ mice to wild-type, we have observed changes over time between the different experimental groups. At the initial stage of disease (4th week of the experimental period), an increased energy expenditure was observed, followed by a transient improvement at the intermediate stage (week 8). However, in the last stage (week 10), the energy expenditure became lower compared to the wild-type group. This reduction can be attributed to reduced activity and fatigue associated with cancer development [43]. These data are consistent with previous observations in humans, in which energy expenditure in advanced gastric and colorectal cancers was not enhanced [42]. Initial RQ values were close to 0.7 in all the experimental groups, and all groups experienced a small increase in RQ with time. However, the RQ increase of the group C2 was greater in the last measurement and showed significant differences with the wild-type and E2 groups. The increase in the C2 group suggests higher oxidation of carbohydrates (RQ ≈ 1) at the expense of fats (RQ ≈ 0.7). Supplemented diets (groups E1 and E2) maintained RQ values similar to the wild-type (C1 group). The increase observed in group C2 may be attributed to enhanced glycolysis in tumor cells to maintain high proliferative rates and a loss of mitochondrial respiratory capacity [44,45,46]. Although Stewart et al. [47] reported that quercetin does not affect the RQ in wild-type animals, in the present case, the group that received microencapsulated probiotics and quercetin (E2 group) showed RQ values more similar to the wild-type (C1 group).

Regarding the microbiota present in the gut of the different experimental groups, our results indicate that dietary supplementation with microencapsulated probiotics and quercetin increased *Bifidobacterium* and *Lactobacillus* populations in feces. These microorganisms are considered beneficial for intestinal function [48] and have also demonstrated preventive effects against the development of chemically-induced colorectal cancer in animal models [49,50]. In the present study, we have observed a reduction of the *Enterobacteriaceae* count, which is a family of Gram-negative bacteria with strong pro-inflammatory potential [51]. The relationship between microbiota and polyphenols is complex and remains mainly unknown. It is known that microbiota plays an important role in the metabolism of polyphenols [52,53]. For example, *Lg* increases the bioavailability of polyphenols when administered together [54]. Our results show that quercetin could modulate intestinal microbiota by stimulating the growth of beneficial microorganisms like *Bifidobacterium* and *Lactobacillus*. Tzounis et al. [55] described a similar effect of polyphenols on intestinal microbiota, boosting the growth of beneficial bacteria like *Bifidobacterium* and inhibiting pathogenic microorganisms. 

Although both supplemented diets used in the present study reduced the number of ACF and adenomas in the colon, a supplemented diet with microencapsulated *Bf, Lg,* and quercetin was more effective. *Bf* and *Lg* reduce intestinal tumorigenesis through suppressed colonic mucosa cellular proliferation [56,57]. Meanwhile, quercetin reduces ACF formation by suppressing the expression of inflammatory mediators [13,58]. The greatest effect of supplemented diet with microencapsulated probiotics and quercetin may be explained by the fact that probiotics may have a greater effect when administered in conjunction with other products [25,56]. Gastrointestinal bleeding is a sign usually associated with colorectal cancer, and prolonged bleeding can cause anemia. The wild-type group (C1) did not show FOB at any time, whereas all Apc^Min/+^ mice showed blood in stool samples in the 1st and 9th weeks of the experimental trial, which is in accordance with other authors [25]. The supplemented diets (E1 and E2 groups) reduced intestinal bleeding when compared to the non-supplemented group. This reduction could be related to the suppressor effect of colonic lesions by supplemented diets.

The Wnt/β-catenin signaling pathway is involved in cell fate determination, control of cell movement, and tissue polarity [59]. In intestinal epithelial cells, mutations that promote constitutive activation of this signaling pathway lead to polyp formation and cancer development. Indeed, the Wnt/β-catenin pathway is constitutively active in most colorectal cancers [27], where the loss of function mutation and/or epigenetic silencing of negative regulators like *APC, AXIN2, DKK1, or SFRP2* are common facts [59]. Quercetin has been described as a potent inhibitor of β-catenin/Tcf signaling in SW480 colon cancer cells and to induce axial defects in Xenopus embryos through inhibition of the Wnt/β-catenin signaling pathway [60]. Besides, long term dietary consumption of probiotics (24 weeks of *B. longum* and *L. gasseri*; 32 weeks of *L. acidophilus* and *B. bifidum*) has been described to reduce the number of DMH-induced ACF in rodents and to protect against the development of CRC in DMH-rat and mouse models [61]. In the present study, we show that 10 weeks of dietary supplementation, either with *Bf* and *Lg* or with a combination of these probiotics plus quercetin, modulated the colonic expression of a specific subset of genes of the Wnt/β-catenin signaling pathway and decreased ACF and tumor numbers in the colon of Apc^Min/+^ mice (Table 6 and Table 7; Figure 6).

A truncated Apc allele is the main genetic characteristic of the Apc^Min/+^ mice. In this animal model, loss of the wild-type Apc allele triggers the formation of polyps, which accumulate high levels of β-catenin [62]. Consequently, we detected both an increased number of ACF (Table 5) and a reduction in the mRNA level of *Cnntb1*, the *β-catenin* gene, in the colon of the Apc^Min/+^ animals (Table 6). Interestingly, Apc^Min/+^ mice fed with supplemented diets displayed β-catenin mRNA levels similar to those of the wild-type animals. These results suggest that dietary supplementation with either probiotics or with probiotics plus quercetin influences the activity of the β-catenin. Analyses of the expression profile of genes involved in Wnt/β-catenin signaling in colon samples of Apc^Min/+^ mice fed either with standard or with supplemented diets further support this idea. In fact, several Wnt/β-catenin signaling antagonists, including *Apc, Daam1, Fbxw4, Nkd1, Ppp2ca, Sfrp2,* and *Tle2*, were downregulated in Apc^Min/+^ mice as compared to wild-type mice (Table 6; Figure 6A). Interestingly, all these Wnt/β-catenin signaling inhibitors recovered either fully or partly their expression level when mice were fed with the supplemented diets. Moreover, most of them increased their expression level more than twice after dietary intervention (Table 7; Figure 6B) while variations in other genes of this signaling pathway were smaller (*Jun*) or, when bigger, the gene expression remained too low to consider its modification physiologically relevant (*Fzd2, Wnt5b*). These results suggest that besides the Apc truncation, lack of other Wnt/β-catenin signaling inhibitors contribute to the modulation of β-catenin in the colon of Apc^Min/+^ mice; and also that the diets supplemented with either probiotics or probiotics plus quercetin exerted their protective action against colon cancer development by inhibiting the canonical Wnt/β-catenin signaling pathway, thus reducing β-catenin degradation and regulating microtubule stabilization. Moreover, although both supplemented diets were effective against the development of ACF and tumors, our results suggest a greater effect for the combination of probiotics and quercetin than for probiotics by themselves.

**Figure 7:**
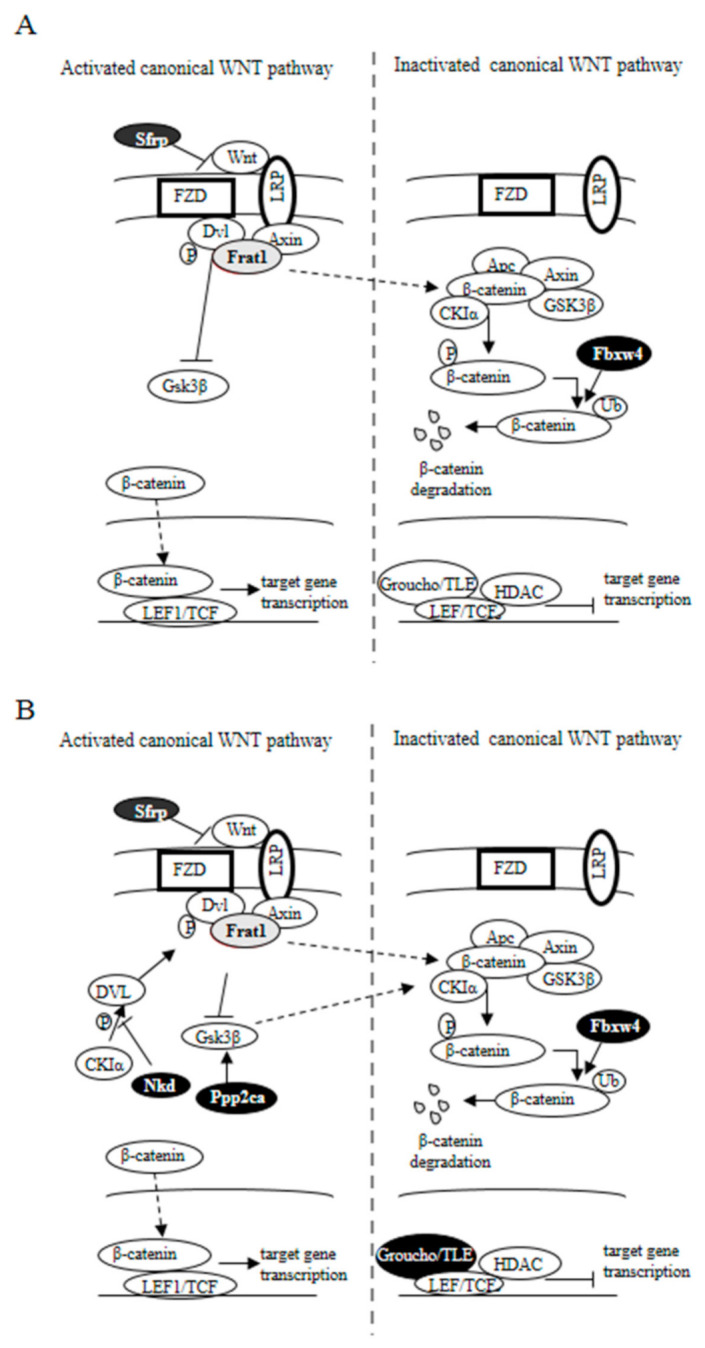
Effect of supplemented diets on the Wnt signaling pathway in APC^Min/+^ mice. Genes whose expression augmented as a result of dietary supplementation are represented with black-filled ovals, while those whose expression decreased are represented in grey. (**A**) APC^Min/+^ mice fed with a (*Bf*+*Lg*) supplemented diet (E1 group). (**B**) APC^Min/+^ mice fed with a (*Bf*+*Lg*+Q) supplemented diet (E2 group). Solid arrow indicates confirmed interaction between proteins; dashed arrow indicates suggested interaction.

In summary, dietary supplementation with microencapsulated probiotics (*Bf* and *Lg*), with or without quercetin, prevented the cancer-related body weight and fat loss, reduced intestinal lesions, and fecal occult blood presence in Apc^Min/+^ mice. Further, the combination of probiotics and quercetin resulted in more effective protection and also prevented hepatomegaly. Mechanistically, our results suggest that microencapsulated probiotics in combination with quercetin could exert an inhibition of the canonical Wnt/β-catenin signaling pathway in the colon of Apc^Min/+^ mice (summarized in Figure 7), which could ultimately be responsible for the observed antitumor efficacy. These aspects raise the importance of more detailed studies to determine the effects of probiotics and dietary flavonoids as a coadjuvant treatment for colorectal cancer. Furthermore, our results indicate that the use of microencapsulation is a useful tool to improve the bioavailability and efficacy of probiotics and dietary bioactive compounds in disease management through modulation of the microbiota.

## 4. Materials and Methods

### 4.1. Probiotics and Quercetin Microencapsulation Procedure

Microcapsules containing *Lg* and *Bf* together with quercetin were prepared as previously described [27]. Briefly, *Lg* and *Bf* and quercetin were incorporated into 10 mL of 20 g/L of sodium alginate. Chitosan aqueous solution was prepared dissolved in 100 mL distilled water acidified with glacial acetic acid to achieve a final chitosan concentration of 0.4% (*w*/*v*). This solution was filtered through a nylon cloth to remove any remaining insoluble material. CaCl_2_ 0.1 M was added to the chitosan solution. The extrusion technique of microencapsulation was performed by using alginate as the supporting matrix. To form beads, the sodium alginate solution was extruded into a previous sterile chitosan solution and stirred. The beads were sieved off from the chitosan solution and washed with sterile distilled water. The capsulates were suspended in a cryoprotectant agent and then frozen at −20 °C. The frozen samples were desiccated under vacuum, at a condenser temperature of −40 °C for about 18 h with a freeze-drier (Freeze-dryer Lyobeta, Telstar, Terrassa, Spain). Dried cells were stored in closed containers at 4 °C, under darkness.

The loading yields for the microencapsulation procedure were 4.07 × 10^9^ CFU/mL for *Lg* and 2.40 × 10^9^ CFY/mL for *Bf*, showing a microencapsulation efficiency of 39.2% for *Lg* and 40.2% for *Bf*. The encapsulation yield was calculated by combining the measurement of the efficacy of entrapment (39.2% for *Lg* and 40.2% for *Bf*) together with the survival of viable cells during the microencapsulation procedure. Surviving bacteria were determined by pour plate counts in MRS agar aerobically incubated at 37 °C for L and in MRS agar anaerobically incubated at 39 °C for B, for 2 days. After 28 days from the preparation of the microencapsulated product, the survival of *Lg* and *Bf* decreased to 3.09 × 10^7^ CFU/mL and 1.38 × 10^7^ CFU/mL, respectively.

Microencapsulated *Lg* and *Bf* were resistant to simulated gastric conditions (pH 2.0, 2 h) and bile solution (3%, 2 h), resulting in significantly (*p* < 0.05) improved survival when compared with free bacteria.

### 4.2. Experimental Animal Model

Five-week-old wild-type C57BL/6J mice (Charles River Laboratories, Barcelona, Spain) and C57BL/6J Apc^Min/+^ mice (Jackson Laboratory, Bar Harbor, ME, USA) were used to test the beneficial effects of a combination of probiotics (*Lg* and *Bf*) and quercetin. The mice, weighing 16–20 g, were housed in cages with filter (2 animals per cage) and kept in a thermostatically controlled room (22 ± 2 °C) at a 12 h light-dark cycle. During 2 weeks, all groups were acclimated and fed with a control standard diet (LabDie®t 5K20, PMI Nutrition International, St. Louis, MI, USA). After the acclimatization period, the following experimental groups were established: Wild-type mice (n = 12) fed a standard diet (LabDiet® 5K20) (Control group, C1). Apc^Min/+^ mice were randomly divided into 3 additional groups (n=12 each) and were fed either a standard diet (Apc^Min/+^ Control group, C2), standard diet supplemented with microencapsulated probiotics (*Lg* and *Bf*, each one 10^7^ CFU/100 g diet) (E1), or a standard diet supplemented with microencapsulated probiotics (*Lg* and *Bf*, each one 10^7^ CFU/100 g diet) plus microencapsulated quercetin (15 mg/100 g diet) (E2). All diets were isonitrogenous and isocaloric. The macronutrient composition of the control standard diet was 18.8% of energy as protein, 54.2% as starch, and 27.9% as fat. Fresh diets were prepared weekly and were kept refrigerated at 4 °C. Food and water were available *ad libitum* during the study. Body weight of all animals was monitored weekly, and food intake was recorded daily. At the end of the 73 days of the experimental period (≈10.5 weeks), mice were anesthetized previously to euthanasia, and different organs (gastrocnemius muscle, spleen, liver, and fat depots such as perirenal, abdominal and epididymal) were extracted, weighed, frozen in liquid nitrogen, and stored at −80 °C.

Animal procedures were performed in accordance with the “Principles of Laboratory Animal Care” formulated by the National Society for Medical Research and the “Guide for the Care and Use of Laboratory Animals” prepared by the Institute of Laboratory Animal Resources, Commission on Life Science, National Research Council, and published by the National Academy Press, revised 1996 [63]. All animal procedures were approved by the Institutional Committee on Care and Use of Laboratory Animals (CEEA, University of Navarra) (Protocol number: CEEA/047-17; approved 25-04-2019).

### 4.3. qPCR Assay

Frozen samples of the distal colon from 6 mice per group, about 45 mg in size, were homogenized using pistils. RNA was extracted using the Illustra RNAspin Mini RNA isolation Kit (GE Healthcare, Buckinghamsire, UK) according to the manufacturer’s instructions. After RNA extraction, cDNA was synthesized from 1µg of total RNA using the RT2 First Strand Kit (SABioscience, Frederick, MD, USA) as described by the manufacturer. The cDNA was then used to detect the expression level of genes involved in Wnt signaling by qPCR using the RT2 Profiler PCR array system specific for the Wnt/β-catenin signaling pathway (Ref.: PAMM-043Z, SA Biosciences). SYBR Green Master Mix (SA Biosciences) was prepared and used according to the manufacturer’s instructions. Briefly, a master mix containing 1µg of cDNA was prepared, and 25 µL of the mix was added to each well of the PCR array. The qPCR was performed in a Chromo 4 thermal cycler (MJ Research, Bio-Rad, Hercules, CA, USA) under the following conditions: 1 cycle of 10 min at 95 °C, to activate the HotStart DNA polymerase, followed by 40 cycles of denaturation at 95 °C for 15 s, annealing at 55 °C for 30 s, and extension at 72 °C for 30 s. SYBR Green fluorescence was measured at every cycle.

### 4.4. Indirect Calorimetry

Measurements of respiratory quotient (RQ) and estimation of energy expenditure (EE) were performed in weeks 4, 8, and 10 of the treatment period. Six mice per group and per time were evaluated. Each animal was placed into the metabolic chamber Oxylet 00 O_2_/CO_2_ indirect calorimeter (Panlab SL, Barcelona, Spain). The oxygen consumption (VO_2_) and carbon dioxide production (VCO_2_) were measured as described [64] during 2 h in sampling periods of 3 min. The recording was completed after 10 sampling periods of date collection. O_2_ consumption and CO_2_ production were used to calculate RQ. EE was estimated according to the Weir equation [65] and related to metabolically active tissue [66]: EE (kcal/day/kg body weight 0.75) = VO_2_ (mL/min) × 1.44 × [3.815 + [1.232 × RQ]].

### 4.5. Faecal Occult Blood

Stool samples were collected from each of the cages at the 1st and 9th weeks of the experimental period. The detection of fecal occult blood (FOB) was immediately assessed by using the Hemoccult® Sensa® test (Beckman Coulter Inc., Brea, CA, USA) following the manufacturer’s instructions and was performed in triplicate for each of the cages (a total of 18 determinations per group). The staining intensities were qualitatively scored by 3 blinded observers. The intensity was estimated as 0 (zero intensity), 1 (low intensity), 2 (moderate intensity), and 3 (high intensity).

### 4.6. Intestinal Microbiota

Samples of stool were collected weekly and used for microbiological analysis. In addition, caecum contents were collected for analysis of *Bifidobacterium* and *Lactobacillus* population. The fresh faecal samples (1g) were suspended in 9 mL of tryptone 0.1% (*w/v*) and homogenized with Stomacher® 3500 (Seward Ltd., West Sussex, UK) during 1 min. Next serial 10-fold dilutions were prepared, after which 1 mL samples of each dilution were plated on: MRS agar (*Bifidobacterium* and *Lactobacillus*), Schaedler agar (total anaerobic microorganism), Reinforced Clostridium agar (*Clostridium*), VRBG (*Enterobacteriaceae*), and VRBL agar (coliform microorganisms). *Lactobacillus* was cultured at 37 °C during 48 h, whereas *Bifidobacterium* were cultured at 39 °C in stove with CO_2_ during 48 h. Total anaerobic microorganisms were incubated in anaerobic jars (AnaeroJar™, Oxoid Ltd., Part of Thermo Fisher Scientific, Hampshire, UK) at 35 °C during 48 h, *Clostridium* were incubated in the same anaerobic jars at 35 °C during 24 h, and *Enterobacteriaceae* and *coliform* microorganisms were incubated at 37 °C during 24 h.

### 4.7. Histological Assessment

The entire colons were removed, rinsed in sterile saline solution (0.9% NaCl), slit open longitudinally, and fixed in formalin solution 10% (*v*/*v*). Colon samples obtained for 4 animals of each group were stained with hematoxylin-eosin. The remaining samples were stained with 0.5% methylene blue solution and examined by transillumination in an optic microscope. Criteria used to identify aberrant crypt foci (ACF) were their increased size, bright blue staining, and flat appearance hidden in the surrounding mucosa.

### 4.8. Statistical Analysis

Body weight data were analyzed by one-way ANOVA test. Food intake and organ weights were analyzed by one-way ANCOVA test with body weight as a covariable. Differences between means were analyzed by Tukey’s post hoc test. A difference at *p* < 0.05 was considered statistically significant. Calculations were carried out using Minitab (v 15, Minitab Inc., State College, PA, USA). A correspondence analysis (CA) to assess the associations between experimental groups and staining intensities was applied to data of faecal occult blood. Data were reformatted into a two-way contingency table prior to applying the CA. The CA was performed with SPAD software (v 7.3. Coeheris, Suresnes, France).

## Figures and Tables

**Table 1:** Body weight (BW), food intake, relative food intake, microencapsulated probiotic, and quercetin intake in the four experimental dietary groups measured at different timepoints.

Variable	C1	C2	E1	E2
Food intake (g/day)				
week 1	3.13 ± 0.28	3.02 ± 0.28	3.05 ± 0.48	2.87 ± 0.35
week 4	2.97 ± 0.31	2.65 ± 0.24	3.11 ± 0.42	3.07 ±0.35
week 8	3.33 ± 0.28	3.07 ± 0.38	3.09 ± 0.48	3.03 ± 0.31
week 10	3.26 ± 0.31	2.60 ± 0.35	2.90 ± 0.35	2.75 ± 0.35
Relative food intake (g/g BW/day))				
week 1	0.13 ± 0.03	0.13 ± 0.01	0.13 ± 0.01	0.12 ± 0.01
week 4	0.12 ± 0.01	0.10 ± 0.01	0.12 ± 0.01	0.12 ± 0.01
week 8	0.12 ± 0.01	0.12 ± 0.01	0.11 ± 0.01	0.11 ± 0.01
week 10	0.11 ± 0.01	0.10 ± 0.01	0.11 ± 0.01	0.10 ± 0.03
Microencapsulated probiotic intake (10^4^ CFU/g BW/day)				
week 1	—	—	2.59	2.43
week 4	—	—	2.38	2.37
week 8	—	—	2.26	2.23
week 10	—	—	2.15	2.09
Quercetin intake (mg/g BW/day)				
week 1	—	—	—	0.018 ± 0.003
week 4	—	—	—	0.018 ± 0.003
week 8	—	—	—	0.017 ± 0.010
week 10	—	—	—	0.016 ± 0.003

Values are mean ± SD, *n* = 12 mice per group. *C1* wild-type mice fed a standard diet, *C2* Apc^Min/+^ fed a standard diet, *E1* Apc^Min/+^ fed a standard diet supplemented with microencapsulated *Bf* and *Lg*, *E2* Apc^Min/+^ fed a standard diet supplemented with microencapsulated *Bf*, *Lg* and quercetin. “—“: Experimental groups not supplemented with probiotics or quercetin.

**Table 2:** Indirect calorimetry in the four experimental groups at different times.

Treatment Time	C1	C2	E1	E2
RQ				
Week 4	0.748 ± 0.022	0.738 ± 0.022	0.738 ± 0.022	0.733 ± 0.020
Week 8	0.764 ± 0.020	0.758 ± 0.015	0.753 ± 0.012	0.761 ± 0.017
Week 10	0.771 ± 0.012 ^b^	0.810 ± 0.015 ^a^	0.789 ± 0.012 ^ab^	0.776 ± 0.020 ^b^
EE (kcal/day/kg^0.75^)				
Week 4	130.4 ± 3.6 ^b^	141.3 ± 3.7 ^a^	143.3 ± 4.0 ^a^	143.8 ± 3.8 ^a^
Week 8	152.9 ± 4.7 ^a^	139.5 ± 3.5 ^b^	152.1 ± 4.0 ^a^	143.9 ± 3.5 ^b^
Week 10	210.8 ± 8.6 ^a^	176.7 ± 4.1 ^b^	177.1 ± 5.2 ^b^	166.5 ± 5.2 ^b^

Values are mean ± SD, *n* = 6 mice per group. Mean values within a row with unlike superscript letters are significantly different (*p* < 0.05) analyzed by one-way ANOVA, followed by Tukey’s post hoc test. *C1* wild-type mice fed a standard diet, *C2* Apc^Min/+^ fed a standard diet, *E1* Apc^Min/+^ fed a standard diet supplemented with microencapsulated *Bf* and *Lg*, *E2* Apc^Min/+^ fed a standard diet supplemented with microencapsulated *Bf*, *Lg,* and quercetin.

**Table 3:** Weight of tissues and organs in the four experimental groups.

Tissue/Organ (g)	C1	C2	E1	E2
Gastrocnemius muscle	0.163 ± 0.014	0.133 ± 0.017	0.154 ± 0.024	0.142 ± 0.014
Liver	1.094 ± 0.128 ^b^	1.216 ± 0.083 ^a^	1.299 ± 0.125 ^a^	1.131 ± 0.225 ^ab^
Spleen	0.063 ± 0.014 ^b^	0.452 ± 0.072 ^a^	0.359 ± 0.097 ^a^	0.400 ± 0.1565 ^a^
Thymus	0.057 ± 0.017	0.032 ± 0.014	0.041 ± 0.021	0.044 ± 0.014
Perirenal fat	0.181 ± 0.097 ^a^	0.058 ± 0.041 ^b^	0.083 ± 0.055 ^b^	0.086 ± 0.055 ^b^
Abdominal fat	0.150 ± 0.111	0.069 ± 0.038	0.120 ± 0.058	0.079 ± 0.038
Epididymal fat	0.658 ± 0.273 ^a^	0.262 ± 0.159 ^b^	0.347 ± 0.183 ^b^	0.329 ± 0.176 ^b^

Value are mean ± SD, *n* = 12 mice per group. Mean values within a row with unlike superscript letters are significantly different (*p* < 0.05) analyzed by one ANCOVA with body weight as covariate and Tuckey’s post hoc test. *C1* wild-type mice fed a standard diet, *C2* Apc^Min/+^ fed a standard diet, *E1* Apc^Min/+^ fed a standard diet supplemented with microencapsulated *Bf* and *Lg*, *E2* Apc^Min/+^ fed a standard diet supplemented with microencapsulated *Bf*, *Lg,* and quercetin.

**Table 4:** Staining intensity frequencies in the four experimental groups analyzed by fecal blood test.

Staining Intensities	C1	C2	E1	E2
Week 1				
0	0	18	6	7
1	0	8	8	8
2	0	9	3	3
3	0	1	1	0
Week 9				
0	1	18	0	3
1	0	4	9	9
2	0	7	6	6
3	0	6	3	0

Data collected for 6 cages per group and 3 replicates per cage. Staining intensity: *0* zero intense, *1* slightly intense, *2* moderate intense, and *3* very intense. *C1* wild-type mice fed a standard diet, *C2* Apc^Min/+^ fed a standard diet, *E1* Apc^Min/+^ fed a standard diet supplemented with microencapsulated *Bf* and *Lg*, *E2* Apc^Min/+^ fed a standard diet supplemented with microencapsulated *Bf*, *Lg,* and quercetin.

**Table 5:** Incidence and counts of aberrant crypt foci and adenomas in the full colon in the four experimental groups.

	C1	C2	E1	E2
Aberrant crypt foci				
Incidence (%)	0	100	100	100
N°/mice	0.00 ^c^	17.75 ^a^	9.75 ^b^	7.50 ^b^
SD	0.00	4.27	4.27	2.51
Adenomas				
Incidence (%)	0	75	50	25
N°/mice	0.00	1.25	0.50	0.25
SD	0.00	1.25	0.25	0.50

Values are mean and SD, *n*= 4 mice per group. Mean values within a row with unlike superscript letters are significantly different (*p* < 0.05). *C1* wild-type mice fed a standard diet, *C2* Apc^Min/+^ fed a standard diet, *E1* Apc^Min/+^ fed a standard diet supplemented with microencapsulated *Bf* and *Lg*, *E2* Apc^Min/+^ fed a standard diet supplemented with microencapsulated *Bf*, *Lg,* and quercetin.

**Table 6:** Fold change in the expression level of genes of the WNT signaling pathway downregulated in APC^Min/+^ mice fed either with a standard diet (C2 group) or with a supplemented diet (E1 and E2 groups) as compared to wild-type mice fed with a standard diet (C1 group).

	WT	APC^Min/+^		
**Downregulated Genes**	**C1**	**C2**	**E1**	**E2**
*Apc*	1	0.493 *	ND	ND
*Csnk1d*	1	0.381 *	ND	ND
*Csnk2a1*	1	0.401 *	0.506 *	ND
*Daam1*	1	0.223 **	ND	ND
*Dixdc1*	1	0.241 **	0.299 *	0.369 *
*Dvl2*	1	0.315 *	0.374 *	ND
*Fbxw2*	1	0.467 *	ND	ND
*Fbxw4*	1	0.314 **	0.543 *	ND
*Fosl1*	1	0.286 *	ND	ND
*Frat1*	1	0.418 *	0.130 **	0.330 *
*Fzd2*	1	0.246 **	0.349 **	-
*Fzd3*	1	0.262 *	0.170 **	0.270 *
*Fzd6*	1	0.395 *	0.259 *	ND
*Lef1*	1	0.161 *	ND	ND
*Lrp5*	1	0.435 *	ND	0.415 *
*Nkd1*	1	0.181 **	0.289 *	ND
*Ppp2ca*	1	0.524 *	ND	ND
*Pygo1*	1	0.405 *	ND	ND
*Sfrp2*	1	0.138 *	ND	ND
*Tle2*	1	0.208 *	ND	ND
*Wisp1*	1	0.249 *	ND	ND
*Wnt11*	1	0.125 **	0.242 *	ND
*Wnt5a*	1	0.349 *	-	ND
*Wnt5b*	1	0.211 **	0.302 *	ND
*Wnt6*	1	0.494 *	0.468 *	ND

Only genes whose expression was downregulated by at least 40% are shown. Data are presented as Mean ± SEM (*n* = 6). Asterisks indicate significant differences among wild-type and APC^Min/+^ mice (*, *p* ≤ 0.05; **, *p* ≤ 0.01). ND: Not detected.

**Table 7:** Fold change in the expression level of genes of the WNT signaling pathway overexpressed or downregulated in APC^Min/+^ mice feed with supplemented diets (E1 and E2 groups) as compared to APC^Min/+^ mice feed with a non-supplemented standard diet (C2).

	APC^Min/+^		
**Upregulated genes**	**C2**	**E1**	**E2**
*Daam1*	1	2.312 *	2.768 *
*Fbxw4*	1	1.729 *	2.272 *
*Fzd2*	1	1.421	3.346 *
*Jun*	1	2.000 **	1.465
*Nkd1*	1	1.595	2.894 *
*Ppp2ca*	1	1.327	2.392 *
*Sfrp2*	1	5.066 *	3.370 *
*Tle2*	1	2.036	4.796
*Wisp1*	1	2.720 *	2.782 *
*Wnt5b*	1	1.428	2.257 *
**Downregulated genes**	**C2**	**E1**	**E2**
*Frat1*	1	0.310 *	0.788

Only genes upregulated more than 2-fold or downregulated by at least 50% are shown. Data are presented as Mean ± SEM (n = 6). Asterisks indicate significant differences among APC^Min/+^ mice fed with standard (control) or supplemented diets (*, *p* ≤ 0.05; **, *p* ≤ 0.01).

## References

[1] Ferlay J., Shin H.-R., Bray F., Forman D., Mathers C., Parkin D.M. (2010). Estimates of worldwide burden of cancer in 2008: GLOBOCAN 2008. Int. J. Cancer.

[2] Ferlay J., Steliarova-Foucher E., Lortet-Tieulent J., Rosso S., Coebergh J., Comber H., Forman D., Bray F. (2013). Cancer incidence and mortality patterns in Europe: Estimates for 40 countries in 2012. Eur. J. Cancer.

[3] Larsson S.C., Wolk A. (2006). Meat consumption and risk of colorectal cancer: A meta-analysis of prospective studies. Int. J. Cancer.

[4] Sesink A.L.A., Termont D.S.M., Kleibeuker J.H., van der Meer R. (2000). Red meat and colon cancer: Dietary haem, but not fat, has cytotoxic and hyperproliferative effects on rat colonic epithelium. Carcinogenesis.

[5] Tisdale M.J. (2009). Mechanisms of Cancer Cachexia. Physiol. Rev..

[6] Yavuzsen T., Walsh D., Davis M.P., Kirkova J., Jin T., Legrand S., Lagman R., Bicanovsky L., Estfan B., Cheema B. (2009). Components of the anorexia–cachexia syndrome: Gastrointestinal symptom correlates of cancer anorexia. Support. Care Cancer.

[7] Tisdale M.J. (1999). Wasting in Cancer. J. Nutr..

[8] Dahm C.C., Keogh R.H., Spencer E.A., Greenwood D.C., Key T.J., Fentiman I.S., Shipley M.J., Brunner E.J., Cade J.E., Burley V.J. (2010). Dietary Fiber and Colorectal Cancer Risk: A Nested Case-Control Study Using Food Diaries. J. Natl. Cancer Inst..

[9] Dihal A.A., de Boer V.C.J., van der Woude H., Tilburgs C., Bruijntjes J.P., Alink G.M., Rietjens I.M.C.M., Woutersen R.A., Stierum R.H. (2006). Quercetin, but not its glycosidated conjugate rutin, inhibits azoxymethane-induced colorectal carcinogenesis in F344 rats. J. Nutr..

[10] Kyle J.A.M., Sharp L., Little J., Duthie G.G., McNeill G. (2009). Dietary flavonoid intake and colorectal cancer: A case–control study. Br. J. Nutr..

[11] Theodoratou E., Kyle J., Cetnarskyj R., Farrington S.M., Tenesa A., Barnetson R., Porteous M., Dunlop M., Campbell H. (2007). Dietary Flavonoids and the Risk of Colorectal Cancer. Cancer Epidemiol. Biomark. Prev..

[12] Kuo S.-M. (1996). Antiproliferative potency of structurally distinct dietary flavonoids on human colon cancer cells. Cancer Lett..

[13] Miyamoto S., Yasui Y., Ohigashi H., Tanaka T., Murakami A. (2010). Dietary flavonoids suppress azoxymethane-induced colonic preneoplastic lesions in male C57BL/Ksj-db/db mice. Chem. Interact..

[14] Oliva J., Bardag-Gorce F., Tillman B., French S.W. (2011). Protective effect of quercetin, EGCG, catechin and betaine against oxidative stress induced by ethanol in vitro. Exp. Mol. Pathol..

[15] Deschner E.E., Ruperto J., Wong G., Newmark H.L. (1991). Quercetin and rutin as inhibitors of azoxymethanol-induced colonic neoplasia. Carcinogenesis.

[16] Murphy E.A., Davis J.M., McClellan J.L., Carmichael M.D. (2011). Quercetin’s Effects on Intestinal Polyp Multiplicity and Macrophage Number in the ApcMin/+ Mouse. Nutr. Cancer.

[17] Ruiz M.J., Fernández M., Picó Y., Mañes J., Asensi M., Carda C., Asensio G., Estrela J.M. (2009). Dietary Administration of High Doses of Pterostilbene and Quercetin to Mice Is Not Toxic. J. Agric. Food Chem..

[18] Fotiadis C.I., Stoidis C.N., Spyropoulos B.G., Zografos E.D. (2008). Role of probiotics, prebiotics and synbiotics in chemoprevention for colorectal cancer. World J. Gastroenterol..

[19] Rafter J., Bennett M., Caderni G., Clune Y., Hughes R., Karlsson P.C., Klinder A., O’Riordan M., O’Sullivan G.C., Pool-Zobel B. (2007). Dietary synbiotics reduce cancer risk factors in polypectomized and colon cancer patients. Am. J. Clin. Nutr..

[20] Carroll I.M., Andrus J.M., Bruno-Barcena J.M., Klaenhammer T.R., Hassan H.M. (2007). Threadgill DS (2007) Anti-inflammatory properties of Lactobacillus gasseri expressing manganese superoxide dismutase using the interleukin 10-deficient mouse model of colitis. Am. J. Physiol Gastrointest. Liver Physiol..

[21] You H.J., Oh D.-K., Ji G.E. (2004). Anticancerogenic effect of a novel chiroinositol-containing polysaccharide from Bifidobac-terium bifidum BGN4. FEMS Microbiol Lett..

[22] Chapman C.M.C., Gibson G.R., Rowland I. (2011). Health benefits of probiotics: Are mixtures more effective than single strains?. Eur. J. Nutr..

[23] Zhou J., Shu Q., Rutherfurd K., Prasad J., Gopal P., Gill H. (2000). Acute oral toxicity and bacterial translocation studies on potentially probiotic strains of lactic acid bacteria. Food Chem. Toxicol..

[24] Graf B.A., Ameho C., Dolnikowski G.G., Milbury P.E., Chen C.-Y., Blumberg J.B. (2006). Rat Gastrointestinal Tissues Metabolize Quercetin. J. Nutr..

[25] Urbanska A.M., Bhathena J., Martoni C., Prakash S. (2009). Estimation of the Potential Antitumor Activity of Microencapsulated Lactobacillus acidophilus Yogurt Formulation in the Attenuation of Tumorigenesis in Apc(Min/+) Mice. Dig. Dis. Sci..

[26] Marteau P., Minekus M., Havenaar R., Huis In’t Veld J.H.J. (1997). Survival of lactic acid bacteria in a dynamic model of the stomach and small intestine: Validation and the effects of bile. J. Dairy Sci..

[27] Chávarri M., Marañón I., Ares R., Ibáñez F.C., Marzo F., Villarán M.D.C. (2010). Microencapsulation of a probiotic and prebiotic in alginate-chitosan capsules improves survival in simulated gastro-intestinal conditions. Int. J. Food Microbiol..

[28] Zhu Y., Luo T.M., Jobin C., Young H.A. (2011). Gut microbiota and probiotics in colon tumorigenesis. Cancer Lett..

[29] Corpet D.E., Pierre F. (2005). How good are rodent models of carcinogenesis in predicting efficacy in humans? A systematic review and meta-analysis of colon chemoprevention in rats, mice and men. Eur. J. Cancer.

[30] Shoemaker A.R., A Gould K., Luongo C., Moser A.R., Dove W.F. (1997). Studies of neoplasia in the Min mouse. Biochim. Biophys. Acta.

[31] Moser A.R., Mattes E.M., Dove W.F., Lindstrom M.J., Haag J.D., Gould M.N. (1993). ApcMin, a mutation in the murine Apc gene, predisposes to mammary carcinomas and focal alveolar hyperplasias. Proc. Natl. Acad. Sci. USA.

[32] Yekkala K., Baudino T.A. (2007). Inhibition of intestinal polyposis with reduced angiogenesis in ApcMin/+ mice due to de-creases in c-myc expression. Mol Cancer Res..

[33] (1993). Guide to the Care and Use of Experimental Animals.

[34] Cordero P., Campion J., Milagro F., Marzo F., Martinez J. (2008). Fat-to-glucose interconversion by hydrodynamic transfer of two glyoxylate cycle enzyme genes. Lipids Heal. Dis..

[35] Weir J.D.V. (1949). New methods for calculating metabolic rate with special reference to protein metabolism. J. Physiol..

[36] Garcia I., de Oteyza C.P., Calvo E., Castuera A. (2007). Alteraciones de la nutrición en Medicina Interna. Análisis de la composición corporal por impedancia bioeléctrica. Rev. Clin. Esp..

[37] Bosaeus I., Daneryd P., Lundholm K. (2002). Dietary Intake, Resting Energy Expenditure, Weight Loss and Survival in Cancer Patients. J. Nutr..

[38] Ockenga J., Valentini L. (2005). Review article: Anorexia and cachexia in gastrointestinal cancer. Aliment. Pharmacol. Ther..

[39] Siddiqui R.A., Hassan S., Harvey K.A., Rasool T., Das T., Mukerji P., DeMichele S. (2009). Attenuation of proteolysis and muscle wasting by curcumin c3 complex in MAC16 colon tumour-bearing mice. Br. J. Nutr..

[40] Tanaka Y., Eda H., Tanaka T., Udagawa T., Ishikawa T., Horii I., Ishitsuka H., Kataoka T., Taguchi T. (1990). Experimental cancer cachexia induced by transplantable colon 26 adenocarcinoma in mice. Cancer Res..

[41] Okuyama T., Oya M., Ishikawa H. (2001). Isolated splenic metastasis of sigmoid colon cancer: A case report. Jpn. J. Clin. Oncol..

[42] You S., Ohmori M., Peña M.M.O., Nassri B., Quiton J., Al-Assad Z.A., Liu L., Wood P.A., Berger S.H., Liu Z. (2006). Developmental abnormalities in multiple proliferative tissues of ApcMin/+ mice. Int. J. Exp. Pathol..

[43] Albert M., Kiefer M.V., Sun W., Haller D., Fraker D.L., Tuite C.M., Stavropoulos S.W., Mondschein J.I., Soulen M.C. (2010). Chemoembolization of colorectal liver metastases with cisplatin, doxorubicin, mitomycin C, ethiodol, and polyvinyl alcohol. Cancer.

[44] Suzuki R., Kohno H., Yasui Y., Hata K., Sugie S., Miyamoto S., Sugawara K., Sumida T., Hirose Y., Tanaka T. (2007). Diet sup-plemented with citrus unshiu segment membrane suppresses chemically induced colonic preneoplastic lesions and fatty liver in male db/db mice. Int. J. Cancer.

[45] Coletta P.L., Müller A.M., Jones E.A., Mühl B., Holwell S., Clarke D., Meade J.L., Cook G.P., Hawcroft G., Ponchel F. (2004). Lymphodepletion in the ApcMin/+ mouse model of intestinal tumorigenesis. Blood.

[46] Fredrix E.W., Soeters P.B., Wouters E.F., Deerenberg I.M., von Meyenfeldt M.F., Saris W.H. (1991). Effect of different tumor types on resting energy expenditure. Cancer Res..

[47] van Norren K., Kegler D., Argiles J.M., Luiking Y., Gorselink M., Laviano A., Arts K., Faber J., Jansen H., van der Beek E.M. (2009). Dietary supplementation with a specific combination of high protein, leucine, and fish oil improves muscle function and daily activity in tumour-bearing cachectic mice. Br. J. Cancer.

[48] Gillies R.J., Robey I., Gatenby R.A. (2008). Causes and Consequences of Increased Glucose Metabolism of Cancers. J. Nucl. Med..

[49] Lopez-Rios F., Sanchez-Arago M., Garcia-Garcia E., Ortega A.D., Berrendero J.R., Pozo-Rodriguez F., Lopez-Encuentra A., Ballestin C., Cuezva J.M. (2007). Loss of the mitochondrial bioenergetic capacity underlies the glucose avidity of carcinomas. Cancer Res..

[50] Wu M., Neilson A., Swift A.L., Moran R., Tamagnine J., Parslow D., Armistead S., Lemire K., Orrell J., Teich J. (2007). Multiparameter metabolic analysis reveals a close link between attenuated mi-tochondrial bioenergetic function and enhanced glycolysis dependency in human tumor cells. Am. J. Physiol. Cell Physiol..

[51] Stewart L.K., Soileau J.L., Ribnicky D., Wang Z.Q., Raskin I., Poulev A., Majewski M., Cefalu W.T., Gettys T.W. (2008). Quercetin transiently increases energy expenditure but persistently decreases circulating markers of inflammation in C57BL/6J mice fed a high-fat diet. Metabolism.

[52] Dolara P., Luceri C., De Filippo C., Femia A.P., Giovannelli L., Caderni G., Cecchini C., Silvi S., Orpianesi C., Cresci A. (2005). Red wine polyphenols influence carcinogenesis, intestinal microflora, oxidative damage and gene expression profiles of colonic mucosa in F344 rats. Mutat. Res. Mol. Mech. Mutagen..

[53] Tuohy K., Rouzaud G., Bruck W., Gibson G. (2005). Modulation of the Human Gut Microflora Towards Improved Health Using Prebiotics—Assessment of Efficacy. Curr. Pharm. Des..

[54] Davis C.D., Milner J.A. (2009). Gastrointestinal microflora, food components and colon cancer prevention. J. Nutr. Biochem..

[55] Jakesevic M., Aaby K., Borge G.-I.A., Jeppsson B., Ahrné S., Molin G. (2011). Antioxidative protection of dietary bilberry, chokeberry and Lactobacillus plantarum HEAL19 in mice subjected to intestinal oxidative stress by ischemia-reperfusion. BMC Complement. Altern. Med..

[56] Bolca S., van de Wiele T., Possemiers S. (2013). Gut metabotypes govern health effects of dietary polyphenols. Curr. Opin. Biotechnol..

[57] Cardona F., Andrés-Lacueva C., Tulipani S., Tinahones F.J., Queipo-Ortuño M.I. (2013). Benefits of polyphenols on gut mi-crobiota and implications in human health. J. Nutr. Biochem..

[58] Tamura M., Ohnishi-Kameyama M., Shinohara K. (2004). Lactobacillus gasseri: Effects on mouse intestinal flora enzyme activity and isoflavonoids in the caecum and plasma. Br. J. Nutr..

[59] Tzounis X., Vulevic J., Kuhnle G.G.C., George T., Leonczak J., Gibson G.R., Kwik-Uribe C., Spencer J.P.E. (2008). Flavanol mon-omer-induced changes to the human faecal microflora. Br. J. Nutr..

[60] Asha A., Gayathri D. (2012). Synergistic impact of Lactobacillus fermentum, Lactobacillus plantarum and vincristine on 1,2-dimethylhydrazine-induced colorectal carcinogenesis in mice. Exp. Ther. Med..

[61] Foo N.-P., Yang H.O., Chiu H.-H., Chan H.-Y., Liao C.-C., Yu C.-K., Wang Y.-J. (2011). Probiotics Prevent the Development of 1,2-Dimethylhydrazine (DMH)-Induced Colonic Tumorigenesis through Suppressed Colonic Mucosa Cellular Proliferation and Increased Stimulation of Macrophages. J. Agric. Food Chem..

[62] Warren C.A., Paulhill K.J., Davidson L.A., Lupton J.R., Taddeo S.S., Hong M.Y., Carroll R.J., Chapkin R.S., Turner N.D. (2009). Quercetin may suppress rat aberrant crypt foci formation by suppressing inflammatory mediators that influence prolif-eration and apoptosis. J. Nutr..

[63] Katoh M., Katoh M. (2007). WNT signaling pathway and stem cell signaling network. Clin. Cancer Res..

[64] Amado N., Fonseca B., Cerqueira D., Reis A., Simas A., Kuster R., Mendes F., Abreu J. (2013). Effects of Natural Compounds on Xenopus Embryogenesis: A Potential Read Out for Functional Drug Discovery Targeting Wnt/β-catenin Signaling. Curr. Top. Med. Chem..

[65] Mohania D., Kansal V.K., Kruzliak P., Kumari A. (2014). Probiotic Dahi containing Lactobacillus acidophilus and Bifidobac-terium bifidum modulates the formation of aberrant crypt foci, mucin-depleted foci, and cell proliferation on 1,2-dimethylhydrazine-induced colorectal carcinogenesis in Wistar rats. Rejuvenation Res..

[66] Batlle E., Henderson J.T., Beghtel H., van den Born M.M.W., Sancho E., Huls G., Meeldijk J., Robertson J., van de Wetering M., Pawson T. (2002). Β-catenin and tcf mediate cell positioning in the intestinal epithelium by controlling the expression of ephb/ephrinb. Cell.

